# 18S and 25S Exonuclease Resistant Ribosomal RNA Molecules Are Produced by 5′‐End Modification During TOR Inhibition.

**DOI:** 10.1002/yea.70007

**Published:** 2025-11-06

**Authors:** Miguel A. Rocha, Gowda Bhavani, Jacob Fleischmann

**Affiliations:** ^1^ Research Division Greater Los Angeles VA Healthcare System California Los Angeles USA; ^2^ Department of Medicine David Geffen School of Medicine at UCLA California Los Angeles USA

## Abstract

*Saccharomyces cerevisiae* yeast cells have been shown to produce 18S and 25S ribosomal RNA molecules that are resistant to degradation by exonucleases, which require a 5′ monophosphate for activity. These resistant RNA species accumulate during the diauxic shift, a phase marked by reduced TOR signaling. To further investigate the link between TOR activity and the accumulation of resistant rRNA, we examined the effects of pharmacological TOR inhibition. Treatment with rapamycin, an active TOR suppressor, led to increased levels of resistant 18S and 25S RNA. Importantly, this accumulation was also observed in cells with constitutively active RNA polymerase I (CARA), indicating that the resistant RNA species arise independently of RNA Pol I transcriptional regulation. Similarly, a TOR1‐deleted mutant of *Saccharomyces cerevisiae* produces resistant 18S and 25S rRNA species in a sustained manner. Thiouracil labeling revealed that rRNA molecules generated during the logarithmic growth phase can be converted into the resistant form, suggesting a posttranscriptional modification process. Furthermore, thiouracil uptake assays demonstrated that overall rRNA synthesis decreases during the diauxic phase. Notably, decapping of the resistant rRNAs restored their sensitivity to exonucleases, indicating that the resistance is conferred by 5′ end modifications, likely involving the addition of one or more phosphate groups.

## Summary

- RNA modifications are crucial in the regulation of gene expression and the stability of RNA molecules.
- In this paper, we describe previously discovered rRNA molecules that are produced by the yeast *Saccharomyces cerevisiae* when the cells are exposed to a nutritionally deprived environment.
- Our main goal was to find what causes these rRNA molecules to become resistant to enzyme digestion. We found the presence of extra phosphates at the beginning of both 18S and 25S species. This resistant capacity, observed when the cells are under stress conditions, might allow the organisms to make proteins needed to survive.

## Graphical abstract

*Saccharomyces cerevisiae* produces exonuclease‐resistant rRNA molecules upon TOR inhibition. This phenomenon occurs when cells enter the diauxic phase, upon addition of rapamycin to the culture, or when TOR activity is reduced through deletion of the TOR1 gene. A 5′‐end modification is implicated in this process, as mid‐log phase cells acquire this resistance, as confirmed by thiouracil‐chase experiments.

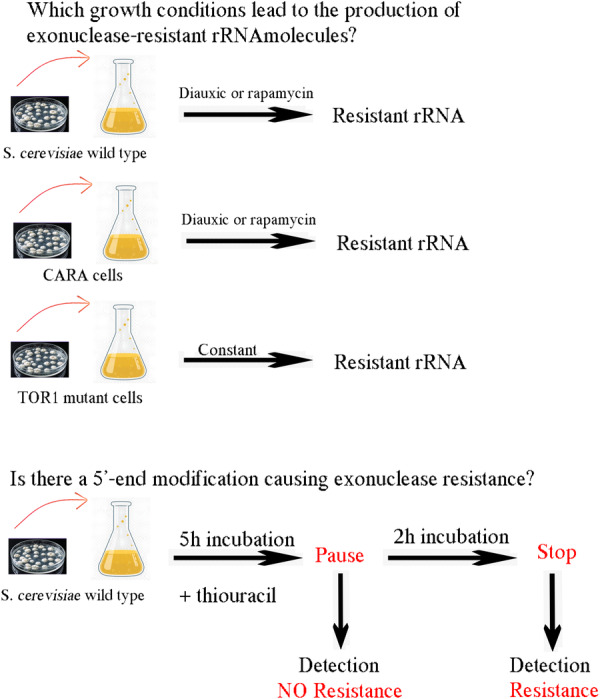

## 1 Introduction

In robustly growing cells, ribosome production consumes the greatest portion of the energy derived from cellular metabolism (Warner 1999). This process is highly sensitive to the availability of nutritional resources and is primarily regulated at the transcriptional level. Three RNA polymerases are utilized in this process (Gutiérrez‐Santiago and Navarro 2023). RNA polymerase I (RNAP I) transcribes multiple repeating ribosomal RNA genes (rDNAs) as 35S precursors, which are processed into 18S, 25S and 5.8S components. RNA polymerase II (RNAP II) transcribes ribosomal protein genes, and RNA polymerase III (RNAP III) copies 5S from the opposite strand of the intergenic sequences of rDNA. Typically, the 18S molecules emerge from the endonucleolytic activity of the processome, and 25S molecules are generated by an endonucleolytic cleavage within ITS2 followed by 5′ to 3′ exonucleolytic trimming. When 18S and 25S are finally processed they have a single phosphate at their 5′end, represented by the phosphate group connecting the 3′ position of one ribose to the 5′ position of the next ribose (Henras et al. 2015). This makes them vulnerable both in vivo and in vitro to attack by 5′ → 3 exonucleases that require 5′‐monophosphates (Jäger et al. 2009). Examples of such nucleases are Terminator (Lucigen) and XrnI (New England Biolabs), which are commercially available for the purpose of degrading rRNA from total RNA. In studies with the pathogenic yeast *Candida albicans*, we unexpectedly found that the cells produced exonuclease‐resistant 18S and 25S molecules (Fleischmann and Rocha 2018). These molecules appeared as the cells approached the diauxic growth phase, with decreasing nutrient availability. We further detected these transcripts in *Saccharomyces cerevisiae*, including in polymerase‐switched cells (Rocha et al. 2022), which lack RNAPI activity.

These resistant molecules have at least one extra phosphate at their 5ʹ end. Treatment with decapping enzymes, like tobacco acid pyrophosphatase(TAP), makes resistant rRNAs susceptible to exonuclease digestion (Lockard et al. 1981). TAP cuts between two phosphates exclusively, thus leaving a single phosphate at the 5ʹ end of these molecule. Having more than one phosphate at the 5ʹ‐end of an RNA molecule is observed in newly transcribed molecules, as RNA polymerases initiate transcription with triphosphate nucleotides (Aprikian et al. 2001). It is unlikely that the resistant 18S and 25S molecules here described are newly transcribed. A modification is more likely to occur during nutritional deprivation, rendering rRNAs resistant to exonuclease degradation. This would provide an advantage to the organisms by supporting ribosome activity despite limited nutritional resources.

The main regulators of cell metabolism in yeast are the Ser/Thr kinases residing in the protein complexes TORC1 and TORC2 (Morozumi and Shiozaki 2021). Complex 1 (TORC1) regulates ribosome biogenesis (Guerra et al. 2022) and translation (Wang et al. 2023), including the transcriptional activity of RNAPI (Sengupta et al. 2010). The current understanding is that TORC1 downregulates the transcriptional activity of RNAPI, RNAPII and RNAPIII in response to dwindling nutrient sources (Sengupta et al. 2010). TOR inhibition also affects the stability of existing ribosomes. For example, the stability of already formed ribosomes is linked to the yeast ribosome preservation factor Stm1, which mediates the formation of nontranslating, dormant 80S ribosomes and protects them from proteasomal degradation (Shetty et al. 2023; Du et al. 2024).

It was of interest to explore any possible role that TORC may play in relation to exonuclease‐resistant 18S and 25S transcripts. We show here that the production of such exonuclease‐resistant 18S and 25S molecules occurs in rapamycin‐suppressed cells and in TORC1‐deleted mutants. Furthermore, we demonstrate that Constitutive Association of Rrn3 and A43 (CARA) cells are capable of accumulating resistant 18S and 25S molecules during diauxic phase and under rapamycin suppression. These CARA cells produce 18S and 25S molecules in a TOR independent manner (Chédin et al. 2007). Additionally, we show that thiouracil‐labeled nascent RNAs transcribed and processed from 35S can be modified to become exonuclease resistant. Altogether, our data suggests a previously undescribed modification of rRNA that confers exonuclease resistance when TOR is fully or partially inhibited.

## 2 Materials and Methods

### 2.1 Organisms

*Saccharomyces cerevisiae* S288C (ATCC), BY28996 (*MAT**a** ura3‐1 leu2‐3, 112trp1‐1 his3‐11, 15 ade2‐1 can1‐100 tor1Δ::KanMX tor2Δ::HIS3 pRS316[TOR2]* (YGRC/NBRP Japan)), W303‐1a (*MATa leu2‐3,112 trp1‐1 can1‐100 ura3‐1 ade2‐1 his3‐11,15)* and FB229‐7A (*MATalpha leu2‐3,112 trp1‐1 can1‐100 ura3‐1 ade2‐1 his3‐11,15, rrn3∆‐:: NAT‐MX4, rpa43∆‐::NAT‐MX4* + *pGEN‐CARA (2micron TRP1 pPGK1‐CARA)* were preserved in 50% glycerol in YPD broth (2% w/V tryptone, 1% w/v yeast extract, 2% w/v dextrose) at −80°C. For activation, cells were inoculated into YPD broth and incubated at 30°C. The yeast culture was maintained on Sabouraud dextrose agar at 4°C and subcultured every 4–6 weeks, with a maximum of five passages. Before experiments, yeast cells were harvested from the agar surface and cultured in YPD broth at 30°C. Cell concentrations were determined using a hemocytometer. Starting yeast concentrations for all experiments were 1 × 10^6 cells/mL. Cultures were incubated during 6 h at 30°C for the Mid log condition and 16 h for the diauxic phase.

### 2.2 Growth Curve Analysis

Yeast cells (*S. cerevisiae* S288C, BY28996, W303‐1a and FB229‐7A) were collected at multiple time points, starting at 4 h and ending at 48 h. Cell concentration was calculated using a hemocytometer. The data were plotted using GraphPad Prism 9 software where each time point represents the average of three experiments. Standard deviation is also shown at each time point.

### 2.3 RNA Isolation

Cells were collected by centrifugation, washed with sterile phosphate‐buffered saline (PBS) and placed on ice until total RNA extraction. Cell disruption was performed using RNase‐free zirconia beads, and RNA was extracted using the RiboPure RNA Purification Kit for Yeast (Invitrogen) following the manufacturer's instructions. RNA concentration and quality were assessed using a Qubit 4 fluorometer and an Agilent 2100 Bioanalyzer.

### 2.4 RNA Analysis

Exonuclease (Terminator or Xrn1) treated and untreated RNA samples were loaded onto an RNA 6000 Nano chip and analyzed using an Agilent 2100 bioanalyzer system (Agilent Technologies, INC). Electropherograms were used to determine the percentage of exonuclease‐resistance RNA by calculating the areas under the peaks of treated (Glynn et al. 2012) RNA and dividing it by the area of untreated RNA (uncut).

### 2.5 Alkaline Phosphatase and Decapping Assays

Cap‐Clip acid pyrophosphatase (CellScript) and alkaline phosphatase calf intestinal (CIP) (New England Biolabs) were used according to the manufacturer's instructions. RNA samples were treated with CIP only, CIP followed by CapClip or CapClip alone. RNAse inhibitors were used to decrease endonuclease activity of CapClip. All samples were purified by ethanol precipitation before proceeding to the exonuclease (Terminator or Xrn1) digestion assay.

### 2.6 5′‐Monophosphate‐Dependent Exonuclease Experiments and 5′‐End Analysis

Total RNA was treated with either Terminator (Biosearch Technologies) or XrnI (New England Biolabs) using the supplied buffer following the manufacturer's protocol. To ensure efficient cleavage, enzymes were applied at a ratio of 1 unit per 1 µg of RNA. RNase inhibitors were included in all assays.

### 2.7 Rapamycin Assays

Yeast cells were incubated with 1 µg of rapamycin (Sigma‒Aldrich) per 1 × 10^6^ cells. The incubation time was 6 h at 30°C with constant shaking. After incubation, the cells were washed with PBS and used for the appropriate assay.

### 2.8 Thiouracil Experiments

Yeast cells were cultured in YPD medium until they reached the desired growth phase, either mid‐log (5 h) or stationary (16 h). Subsequently, 100 uM Thiouracil was added to the culture, and the cells were incubated for an additional hour. Cells were washed in PBS and stored for RNA extraction. For the chase component of the experiment, the cells were spun down, and the YPD medium containing thiouracil was removed. The cells were then resuspended in YPD, which had previously supported the growth of an identical number of yeast cells for the same length of time. Those yeast cells were removed by spinning them down before resuspension.

### 2.9 Detection of Thiouracil by Blotting

Thiouracil (Sigma) incorporation was measured by biotin detection using MTSA‐biotin‐XX (Biotium). Biotinylation reactions included RNA (1 µM), 10 mM HEPES (pH 7.5), 1 mM EDTA, and 25 µM MTS‐biotin (dissolved in DMSO at 250 µM).

RNA was separated on formaldehyde agarose gels (Lonza) and stained with SYBR Gold nucleic acid gel stain (Life Technologies) for 30 min. Gel images were captured with a digital camera (Canon Vixia HFS30). RNA was transferred by electroblotting (Bio‐Rad Trans‐Blot Turbo Transfer System) to a positively charged nylon membrane (Millipore) in 0.5x TBE (standard Tris/Borate/EDTA buffer). The RNA was cross‐linked to the membrane using UV (Stratagene UV Crosslinker). The Prosignal (Prometheus) chemiluminescence substrate was used to detect the HRP signal. The film was developed with an SRX‐101A Konica film processor.

### 2.10 Statistical Analysis

All experiments were performed in triplicate, and data are presented as mean ± standard deviation (SD). Statistical significance was determined using two‐tailed, unpaired *t*‐test in GraphPad Prism (version 9.5, GraphPad Software, San Diego, CA, USA). A *p* < 0.05 was considered statistically significant.

## 3 Results

### 3.1 Production of Resistant 18S and 25S in Wild‐Type Saccharomyces and in TOR‐Deleted Mutants in Relation to Their Growth Phases

**Figure 1:**
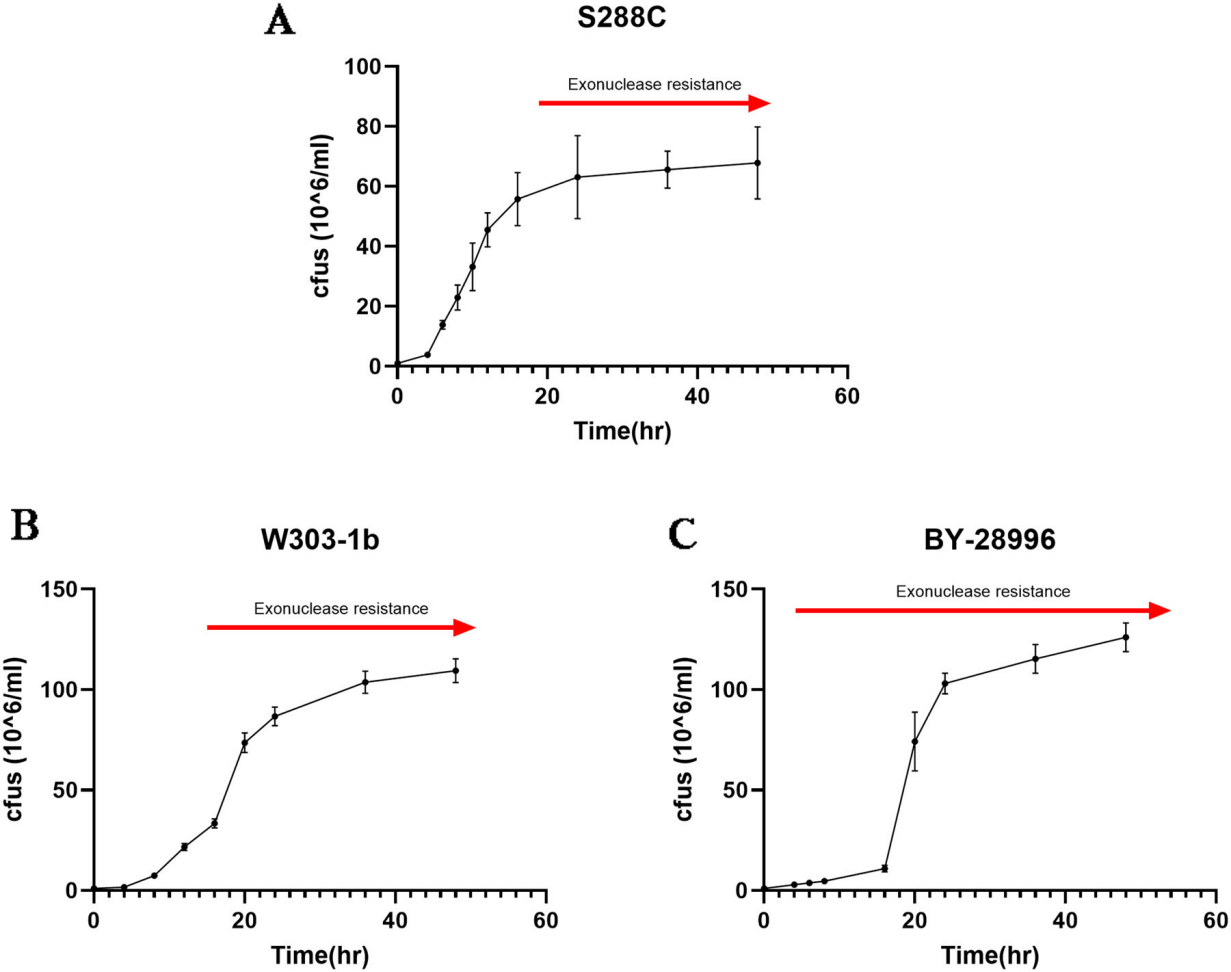
Growth curves of the yeast used in this study: (A) *Saccharomyces cerevisiae* wild type (S288C), (B) W303‐1b and (C) *S. cerevisiae* TOR mutant (BY‐28996). Cells were incubated in YPD medium at 30°C until diauxic phase was reached. Red arrows indicate the time when exonuclease resistant 18S and 25S molecules appear in relation to the yeast growth curve.

**Figure 2:**
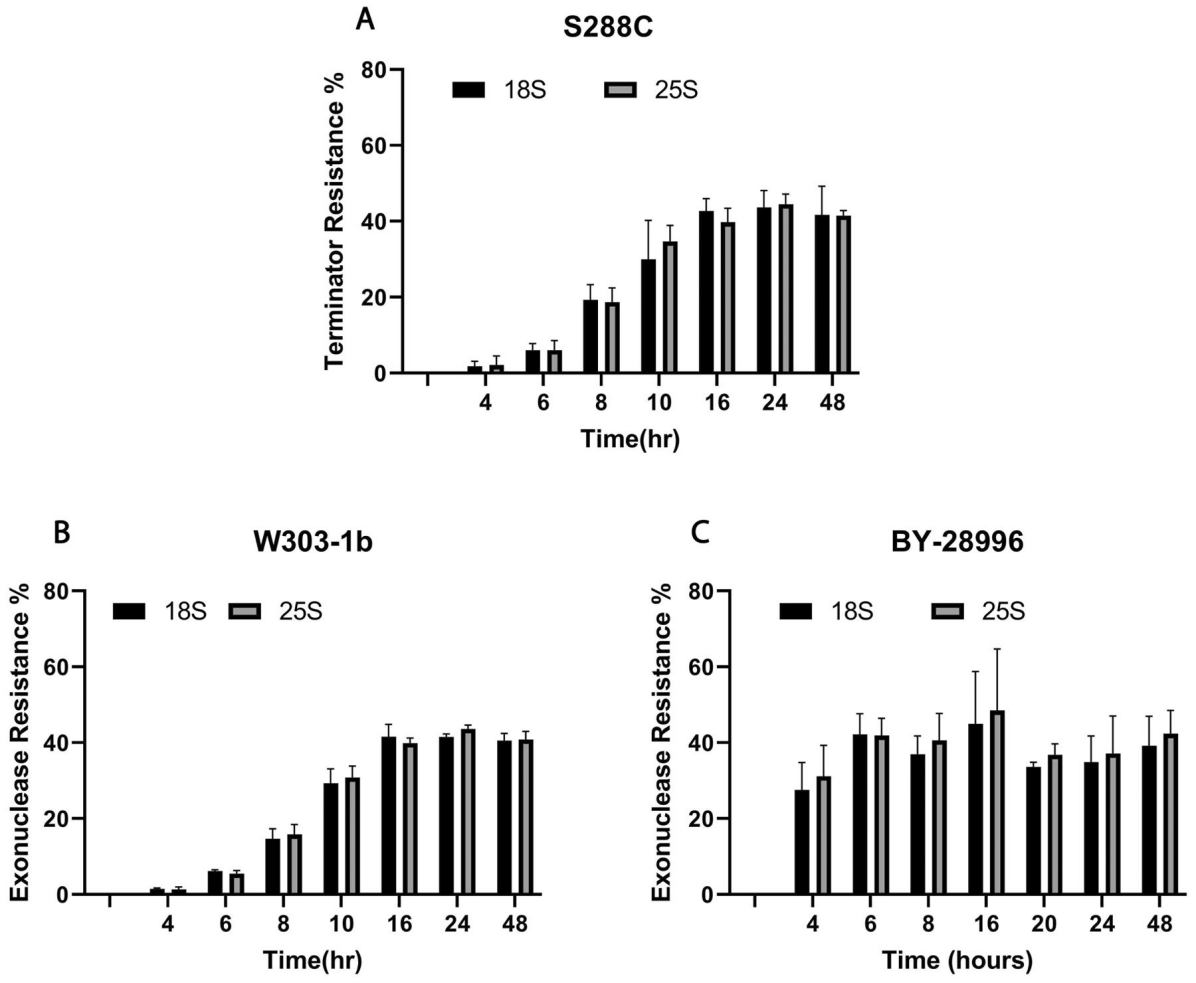
Exonuclease (Terminator or Xrn1) percentage resistance in RNA from (A) *Saccharomyces cerevisiae* wild type S288C, (B) *S. cerevisiae* TOR‐mutant parental cell line W303‐1b and (C) TOR‐mutant BY‐28996. Ratios of cut to uncut ribosomal 18S and 25S were obtained using Agilent Bioanalyzer 2100. Error bars represent standard deviation from three different experiments.

During the growth cycle of yeast, the rate of new yeast formation diminishes as nutrient availability decreases, eventually reaching a diauxic phase. As shown in Figure 1, the growth curve of the TOR1‐mutant S. *cerevisiae* BY‐28996 (Figure 1C) is similar to the wild‐type *Saccharomyces cerevisiae* S288C (Figure 1A) and to the parental cell line W303‐1b (Figure 1B). They all present a lag, logarithmic and diauxic phase, but differ on the timing of these phases. In the wild type cells, production of resistant 18S and 25S is clearly phase dependent, as it increases when cells approach the diauxic phase (Figure 2A,B). TOR1‐deleted BY‐28996 took longer (Figure 1), but in contrast to S288C or W303‐1b cells, the production of resistant 18S and 25S molecules remained constant and reached similar levels throughout all the phases (Figure 1C).

### 3.2 5′‐End Analysis of Exonuclease Resistant 18S and 25S From Wild‐Type Strains at Different Growth Phases

**Figure 3:**
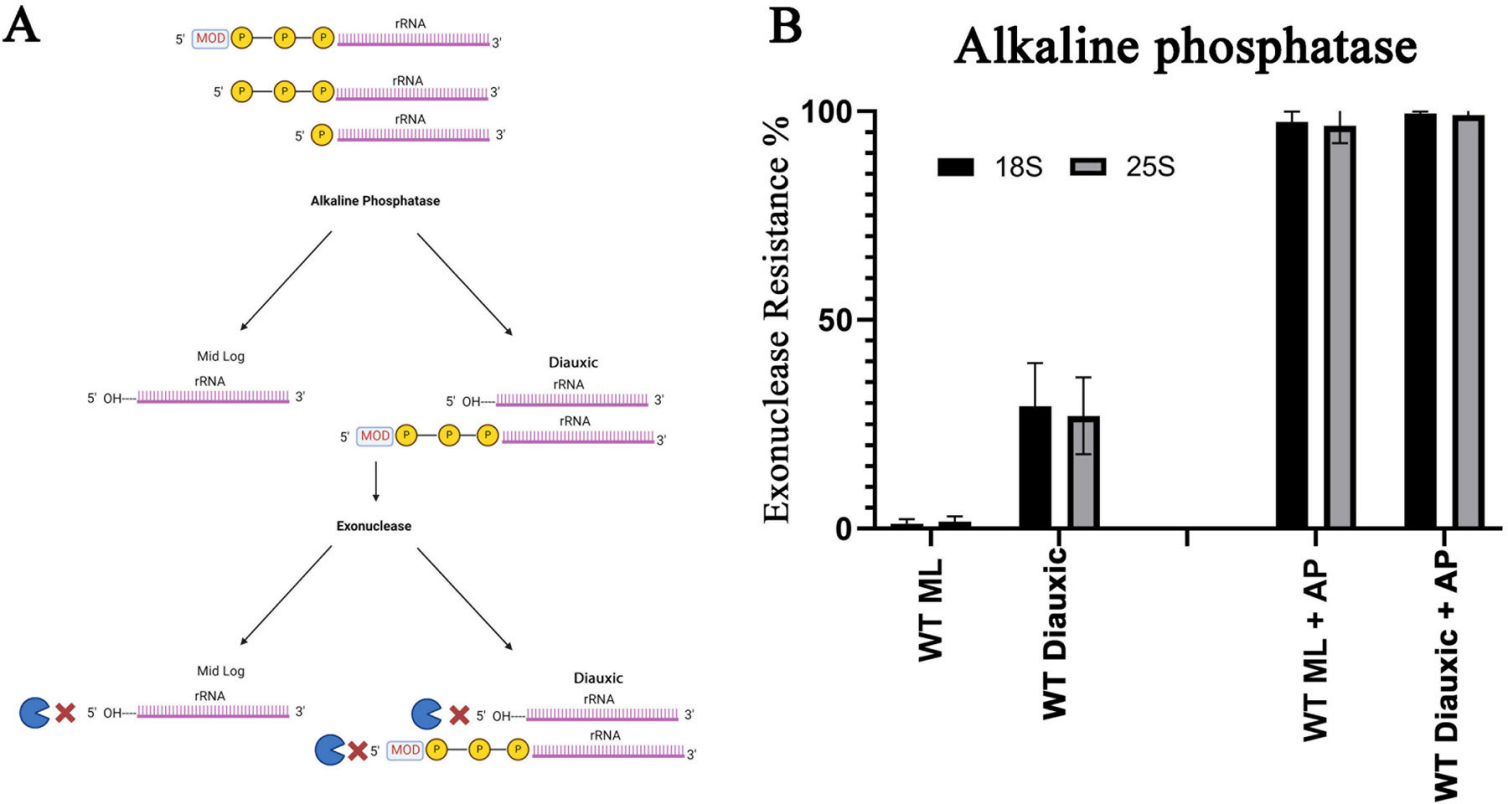
5ʹ end analysis of ribosomal RNA extracted from wild type cells under mid‐log and stationary conditions. (A) Illustration of the possible 5’‐end forms of 18S and 25S and the predicted outcome after enzyme treatment to both mid‐log and stationary organisms. (B) Exonuclease resistance percentages from *Saccharomyces cerevisiae* wild type mid‐log (WT ML) and diauxic (WT diauxic) were calculated after RNA was treated with alkaline phosphatase (AP) or without treatment. Error bars represent standard deviation from three different experiments.

**Figure 4:**
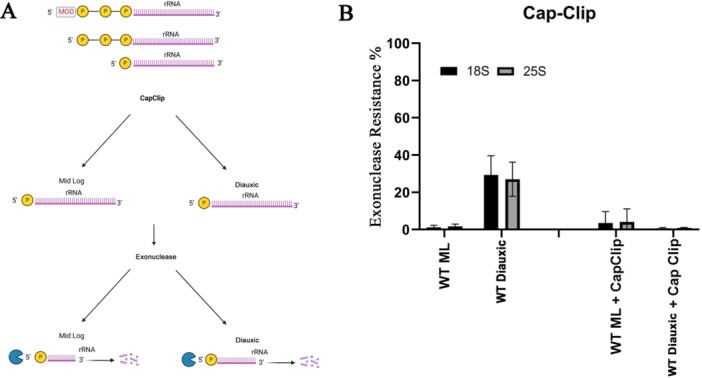
5ʹ end analysis of ribosomal RNA extracted from wild type cells under mid‐log and stationary conditions. (A) Illustration of the possible 5ʹ‐end forms of 18S and 25S and the predicted outcome after enzyme treatment to both mid‐log and stationary organisms. (B) Exonuclease resistance percentages from *Saccharomyces cerevisiae* wild type mid‐log (WT ML) and diauxic (WT diauxic) were calculated after RNA was treated with acid pyrophosphatase (Cap Clip) or without treatment. Error bars represent standard deviation from three different experiments.

**Figure 5:**
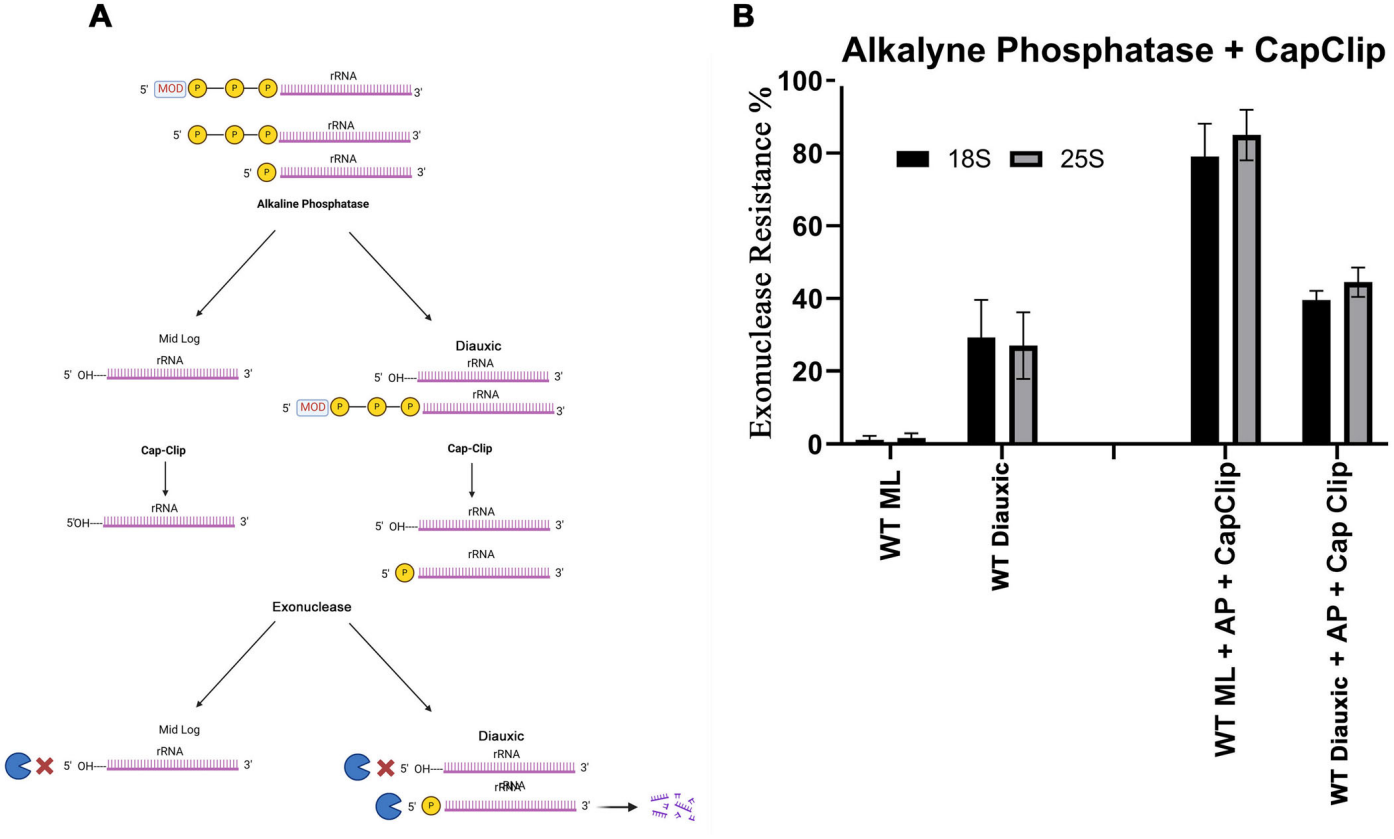
5ʹ end analysis of ribosomal RNA extracted from wild type cells under mid‐log and stationary conditions. (A) Illustration of the possible 5ʹ‐end forms of 18S and 25S and the predicted outcome after enzyme treatment to both mid‐log and stationary organisms. (B) Exonuclease resistance percentages from *Saccharomyces cerevisiae* wild type mid‐log (WT ML) and diauxic (WT Diauxic) were calculated after RNA was treated sequentially with AP and Cap Clip, or without treatment. Error bars represent standard deviation from three different experiments.

As mentioned, normally processed 18S and 25S molecules have a single phosphate at their 5ʹ end and are susceptible to these exonucleases. Thus, for them to become resistant, some changes need to occur. Several factors can make RNA molecules resistant to single 5ʹ‐phosphate‐dependent RNA exonucleases (Figures 3, 4, 5). One possibility is the removal of the single phosphate at its 5ʹ end, leaving a hydroxyl group at the 5ʹ position of the first ribose. Another is the addition of extra phosphates at the 5ʹ end. A third option is the attachment of a nonphosphate structure, such as a cap, to the 5ʹ end phosphate. To identify which of these scenarios is most likely, we employed alkaline phosphatase (AP) (Figure 3), the decapping enzyme Cap‐Clip, which is a pyrophosphatase that cuts only between phosphates (Figure 4), and the combination of both enzymes (Figure 5).

Exonuclease resistance of RNA in the diauxic phase of yeast cells can arise through several mechanisms, as depicted in Figure 3A. One possibility is the presence of multiple phosphates at the 5ʹ end without a cap, which makes rRNA resistant to exonuclease digestion. AP removes these phosphates, yet the rRNA remains resistant to exonuclease. Another possibility is that the resistant molecules undergo structural modifications that confer resistance to both exonucleases and AP. In such cases, even after dephosphorylation, the rRNAs maintain their resistance, with the remaining resistance likely due to AP removing the single phosphate from normally processed molecules.

As shown in Figure 3B, in the mid‐log growth phase of the yeast, essentially all the 18S and 25S molecules were susceptible, indicating that they all contained a single phosphate at the 5ʹ end, as expected for transcribed and processed 18S and 25S molecules. Alkaline phosphatase treatment increases rRNA resistance to 100% by removing the single phosphate and leaving a hydroxyl group at the 5ʹ end. As the cells enter the stationary phase, 30%–40% of the rRNA molecules become resistant to exonucleases, suggesting a modification at their 5ʹ end. When these molecules, from the diauxic phase, are dephosphorylated (AP treatment), their resistance also reaches 100%. Total resistance increase is due to the removal of the single phosphate of previously susceptible rRNA molecules.

Figure 4A depicts the possibilities for Cap‐Clip activity. In the mid‐log phase Cap‐Clip does not modify rRNA since there is a single phosphate at the 5ʹ end. Therefore, exonuclease digestion is the same, with or without Cap‐Clip treatment. In the diauxic phase, the possibilities include single phosphate, triphosphate alone or with a modification, such as a cap. Cap‐Clip digestion leaves a single phosphate in all these possibilities.

When RNA from mid‐log and diauxic cells was treated with Cap‐Clip (Figure 4B), all RNA was digested by exonucleases. This suggests that resistant molecules in the diauxic phaser acquire additional phosphates to evade exonuclease degradation (Figure 4B).

Figure 5A shows the results of sequential treatment with AP and Cap‐Clip. RNA molecules from mid‐log yeast, which are fully susceptible to exonucleases due to their 5ʹ‐monophosphate, become resistant after AP plus Cap‐Clip digestion. The pyrophosphatase activity of Cap‐Clip does not restore susceptibility. AP removes the phosphates, leaving an OH group at the 5ʹ end, rendering the RNA resistant to exonucleases, while Cap‐Clip has no further effect. The observation that diauxic organisms have approximately 35% exonuclease resistance also indicates that approximately 65% of 18S and 25S molecules still carry a single 5ʹ‐phosphate. We observed a small amount of RNase activity when using Cap‐Clip (Figure S2) that accounts for the missing resistance percentage in both midlog and diauxic rRNA (Figure 5B). We know that resistant molecules have more than one phosphate at their 5ʹ end, as they become susceptible after decapping. Since AP was the first enzyme in the sequential digestion, and exonuclease resistance did not reach 100%, the extra phosphates must have been protected from AP by some additional modification at the 5ʹ end. Cap‐Clip makes them susceptible to exonucleases, even with the additional structure. The remaining molecules with 5ʹ‐monophosphates become exonuclease resistant by the initial AP removal of the phosphates.

### 3.3 Effects of Rapamycin Inhibition and TOR1 Deletion on Resistant 18S and 25S

**Figure 6:**
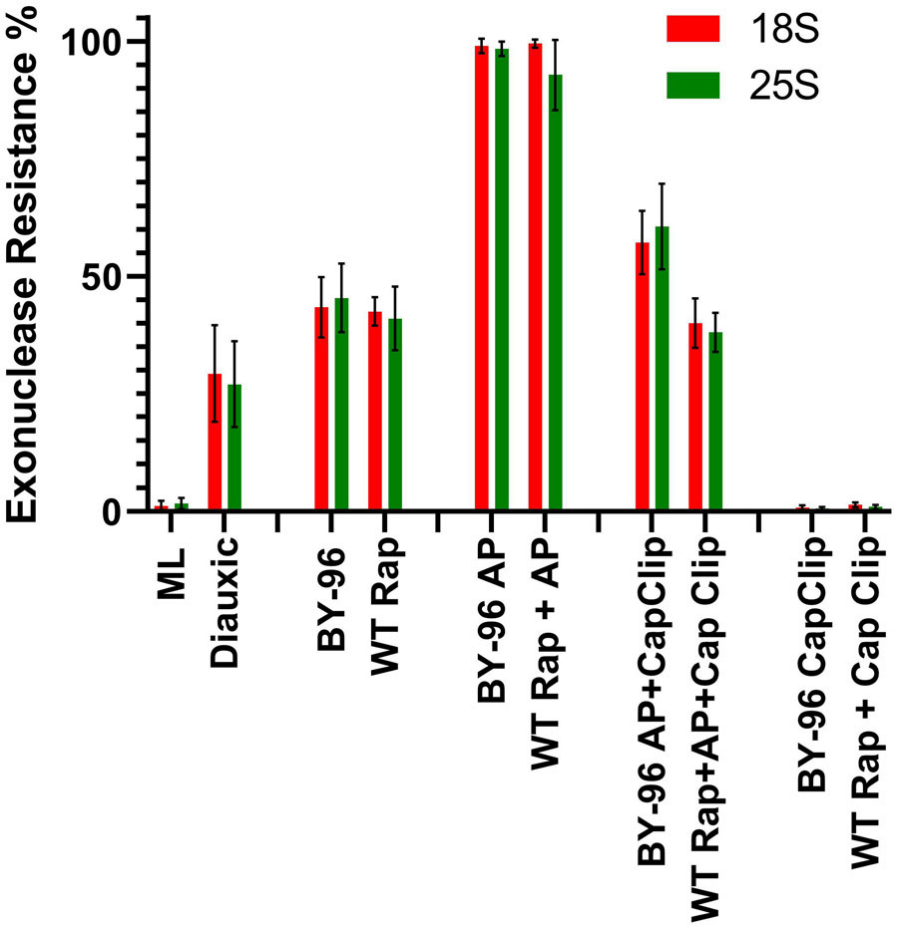
5ʹ end analysis of ribosomal RNA extracted from wild type cells exposed to rapamycin, and from TOR‐mutant cells. Exonuclease resistance percentages from *Saccharomyces cerevisiae* wild type plus rapamycin (WT Rap) and TOR‐mutant cells (BY‐96) were calculated after RNA treatment with alkaline phosphatase (AP), acid pyrophosphatase (Cap Clip), and both AP and Cap Clip. Error bars represent standard deviation from three different experiments.

As shown in Figure 6, both TOR1‐deleted yeast cells (BY‐28996) and wild type (WT) treated with rapamycin produced resistant 18S and 25S as in those produced by wild‐type cells nearing growth inhibition. When BY‐28996 and WT + Rap cells were treated with alkaline phosphatase or decapping pyrophosphatase (Cap‐Clip) individually or in sequence, the results resembled those of wild‐type *S. cerevisiae* (Figures 3, 4, 5) during the diauxic growth phase. Thus, it appears that regardless of the mechanism by which 18S and 25S are converted to an exonuclease‐resistant state, it seems to be suppressed by TOR1.

### 3.4 Role of RNAPI and TOR in the Appearance of Exonuclease Resistant 18S and 25S Molecules

**Figure 7:**
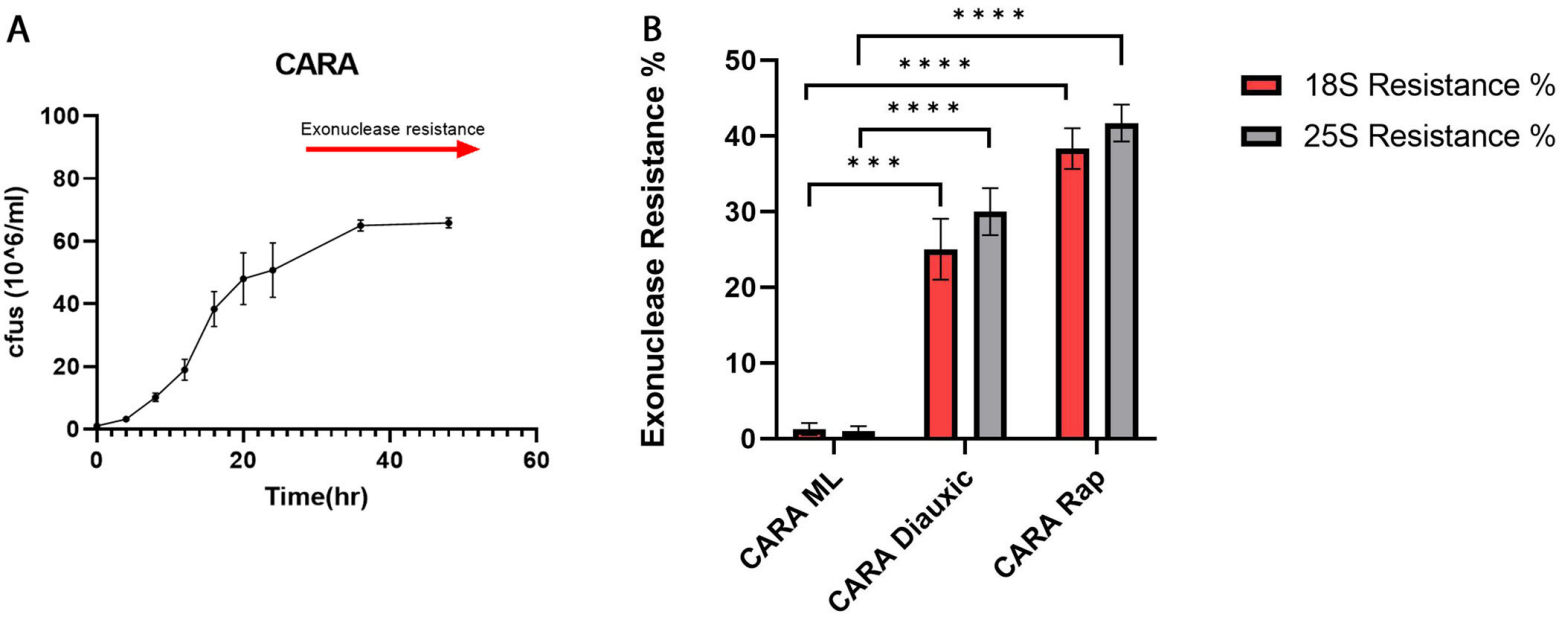
Exonuclease resistance in CARA cells. rRNA 18S and 25S from CARA cells cultured during midlog (ML) and diauxic phases (A) show a significantly different resistance to exonuclease. CARA cells exposed to rapamycin show higher exonuclease resistance percentage when compared to midlog or diauxic cells (B). Error bars correspond to the standard deviation of three independent experiments. Statistical significance was calculated using unpaired *t*‐test, *** *p* < 0.0005 **** *p* < 0.0001.

As our data regarding exonuclease resistance revealed a modification at the 5ʹ ends of resistant 18S and 25S when TOR1 is inactive, we wanted to determine whether this process functions independently of the usual polycistronic transcription by RNAPI and processing of rRNA. To this end, we used CARA cells, where RNAPI is constitutively active, regardless of the TOR pathway status. Figure 7A shows the growth curve of CARA cells indicating onset of exonuclease resistance production (red arrow). Figure 7B shows exonuclease resistance percentages of CARA cells during mid‐log and diauxic phases, and after rapamycin treatment. There is a significant difference (as indicated by the *p* values) between mid‐log CARA cells and diauxic or rapamycin treated cells.

### 3.5 Thiouracil Labeling of rRNA to Assess Timing of 18S and 25S Synthesis and Modification

**Figure 8:**
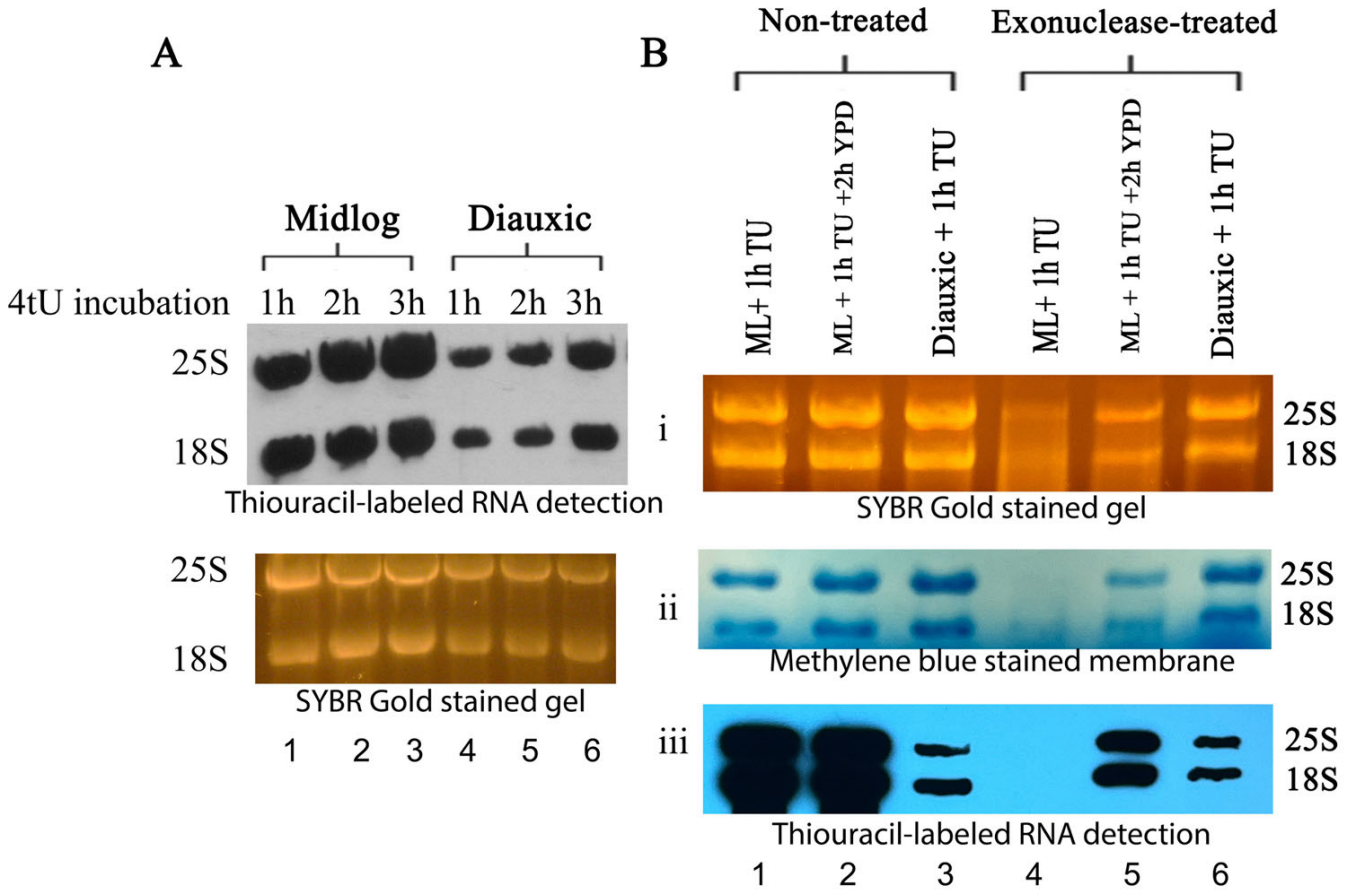
Detection of RNA transcription in *Saccharomyces cerevisiae*. (A) 4‐Thiouracil was added to the cultures after organisms reached midlog or diauxic phase for 1, 2, and 3 h. Thiol‐specific biotinylated RNA (1 µg) was separated on agarose, transferred onto a charged nylon membrane and detected with streptavidin‐HRP. (B) Exonuclease resistance in newly synthesized RNA. Ribosomal RNA from Midlog (ML) and diauxic S. *cerevisiae* grown in the presence of thiouracil was labeled with MTSA‐biotin and separated on a 1.2% agar gel, stained with SYBR‐gold dye (i), transferred onto nitrocellulose membrane followed by methylene blue staining (ii) and finally, the biotinylated RNA was detected by HRP‐Streptavidin (iii) As a way to control for background HRP‐Streptavidin signal, samples without biotin or thiouracil are included in Figure S2.

We used timed thiouracil labeling of nascent RNA combined with exonuclease resistance measurements utilizing gel analysis and bioanalyzer measurements. A preliminary look at thiouracil labeling (Figure 8A) indicated that it reliably reflected the rate of rRNA synthesis, differentiating the mid‐log phase from the diauxic growth phase. This gave us the opportunity to label the rRNAs at a particular phase and monitor them to determine whether these molecules undergo post‐synthesis modifications that confer resistance to exonuclease digestion. As shown in Figure 8B Lane 1, the yeast that were labeled from hours 4–5 in fresh YEPD (their usual mid‐log phase) were completely digested by exonuclease (Lane 4). When yeast that were labeled for the same time period were allowed to rest for another 2 h with the same degree of nutritionally depleted YEPD without thiouracil (approaching the diauxic phase, Lane 2), they became resistant to exonuclease digestion (Lane 5). The fact that they contained thiouracil indicates that they were synthesized between hours 4 and 5, and clearly, some modification occurred to them over the next 2 h, leading to exonuclease resistance. When the yeast cells were incubated overnight (diauxic growth phase) and labeled with thiouracil for 1 h (lane 6), they produced newly synthesized exonuclease‐resistant 18S and 25S molecules. These data indicate that when the 5ʹ‐end modification system is active, both previously synthesized and newly formed ribosomal RNA molecules are targeted for these changes.

## 4 Discussion

We have reported the existence of exonuclease‐resistant ribosomal 18S and 25S RNA molecules in yeast. These molecules are produced consistently as the nutritional support for cell growth diminishes, a phase in which TOR downregulates RNAPI activity. Our data showing the production of these molecules with TOR inhibition by rapamycin indicate that this process is possibly suppressed by TOR. The consistent production of resistant 18S and 25S by TOR‐deleted yeast cells adds additional support for TOR involvement. Thus, there are two possible mechanisms involve in the production of these molecules, one related to TOR downregulation and another one related to RNAPI transcription.

To study this we used CARA cells, which have a constitutively active RNAPI (Figure 7). These cells do not produce resistant molecules during logarithmic growth, but they do in the diauxic phase or when rapamycin is added. This indicates that transcription by RNAPI per se is not adequate in the synthesis of the resistant rRNA molecules, and thus, a modification is required for resistance conversion. Thiouracil labeling of ribosomal RNAs showed that this conversion to exonuclease resistance involves both previously produced and processed molecules, as well as newly transcribed molecules.

The mechanism by which these molecules become exonuclease resistant is unknown. The fact that these processed molecules can be made susceptible to a 5ʹ‐dependent exonuclease by a decapping reaction, indicates that any modification occurring at the 5ʹ‐end that initially makes them resistant to exonuclease digestion, at minimum, includes an added phosphate by a kinase reaction. It is well established for recycling of mRNAs in the cytoplasm that molecules whose triphosphate 5ʹ‐end is lost to a monophosphate state become targets for 5ʹ‐kinases and guanylyltransferases for recovery (Trotman and Schoenberg 2019). It is also well known that the polycistronic ribosomal RNA products of RNAPI transcription are processed to small and large ribosomal RNA components with a single phosphate at their 5ʹ end, making them targets for such kinases (Woolford and Baserga 2013). However, this alone does not explain why rRNA resistance is detected exclusively as the cell approaches the diauxic growth phase and is not detected in the robust mid‐log phase.

Based on the timing of the thiouracil experiments, it is highly likely that resistance conversion occurs in the cytosol. We have also reported that this exonuclease resistance can develop in the nucleus (Fleischmann et al. 2020). This would be different from the capping of mRNA in the nucleus, which results from the removal of the terminal phosphate of the 5ʹ‐triphosphated RNA by RNA triphosphatase, resulting in biphosphate 5ʹ ends, to which a guanylyl triphosphate is added by a transferase with the release of two phosphate molecules.

There are genes in *S. cerevisiae* whose transcription is specifically activated in the diauxic phase, and these genes need to be translated (Riou et al. 1997). Thus, maintaining an adequate number of ribosomes is advantageous for the cell, and protecting 18S and 25S molecules from exonuclease digestion helps with this process. Indeed, such exonuclease‐resistant ribosomal RNAs have been found in ribosomes isolated from *Candida albicans* (Fleischmann et al. 2020). It has also been proposed that yeast that approaches the diauxic growth phase and the phase itself could be a possible model for the study of aging (Werner‐Washburne et al. 2012). The conversion of otherwise susceptible to exonuclease‐resistant ribosomal RNA components that we are describing is a phenomenon specific for the later stage of the yeast cycle, and defining the details of this process could be beneficial in this area of investigation.

## Author Contributions

Jacob Fleischmann conceived and designed the study, analyzed the data, wrote the manuscript, revised and approved final version of the manuscript. Miguel A. Rocha designed and performed the experiments, collected and analyzed the data, wrote materials and methods section. Gowda Bhavani performed the thiouracil experiments, collected and analyzed data related to thiouracil experiments. All authors approved the final version of the manuscript.

## Ethics Statement

The authors have nothing to report.

## Consent

The authors have nothing to report.

## Conflicts of Interest

The authors declare no conflicts of interest.

## Supporting information


**Supplementary Figure 1:** HRP‐Streptavidin background signal. **Supplementary Figure 2:** RNA was treated with three different decapping enzymes: RppH, mRNA decapping enzyme (MDE) and Cap‐Clip to check for endonuclease activity.

## Data Availability

The data that supports the findings of this study are available in the Supporting Information of this article.
