# Supplementary material for: 18S and 25S Exonuclease Resistant Ribosomal RNA Molecules Are Produced by 5′‐End Modification During TOR Inhibition
Source: Yeast. 2025 Nov 6;42(12):273–82. doi: 10.1002/yea.70007 (PMC12757822; doi:10.1002/yea.70007)
Supplement: Supplementary file 1 — Supplementary Figure 1: HRP‐Streptavidin background signal. Supplementary Figure 2: RNA was treated with three different decapping enzymes: RppH, mRNA decapping enzyme (MDE) and Cap‐Clip to check for endonuclease activity. [file YEA-42-273-s001.docx]

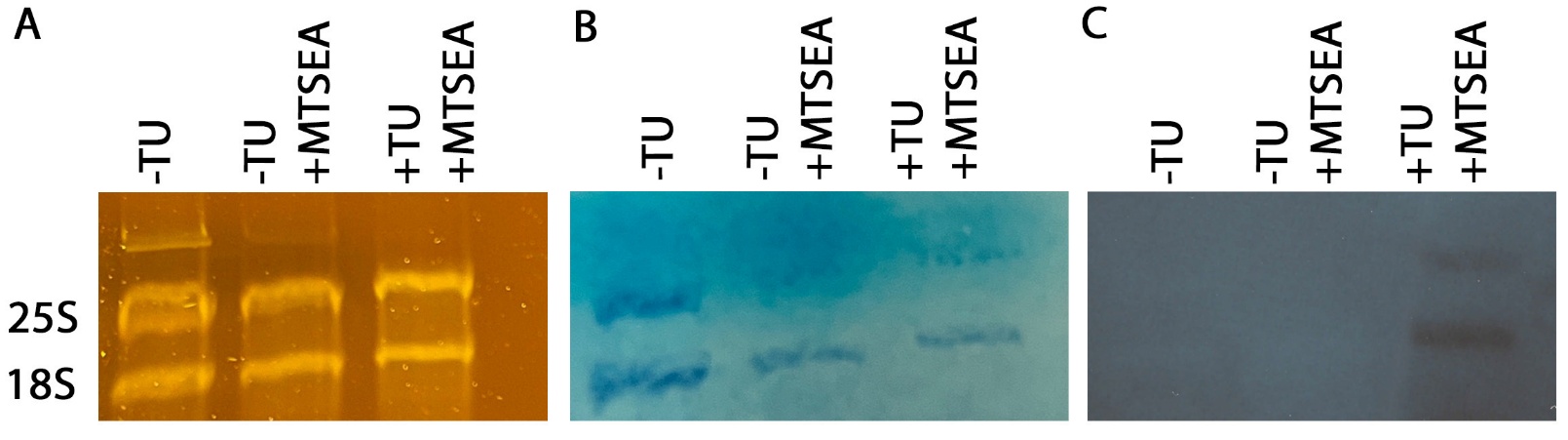


Supplementary Figure 1. HRP-Streptavidin background signal. (A) SYBR-Gold stained gel showing equal amounts of total RNA for each condition: RNA without thiouracil (-TU), RNA without thiouracil plus MTSEA-biotin (-TU+MTSEA) and RNA with thiouracil plus MTSEA-biotin (+TU+MTSEA). A methylene stained membrane is shown in (B) and the developed film in (C).


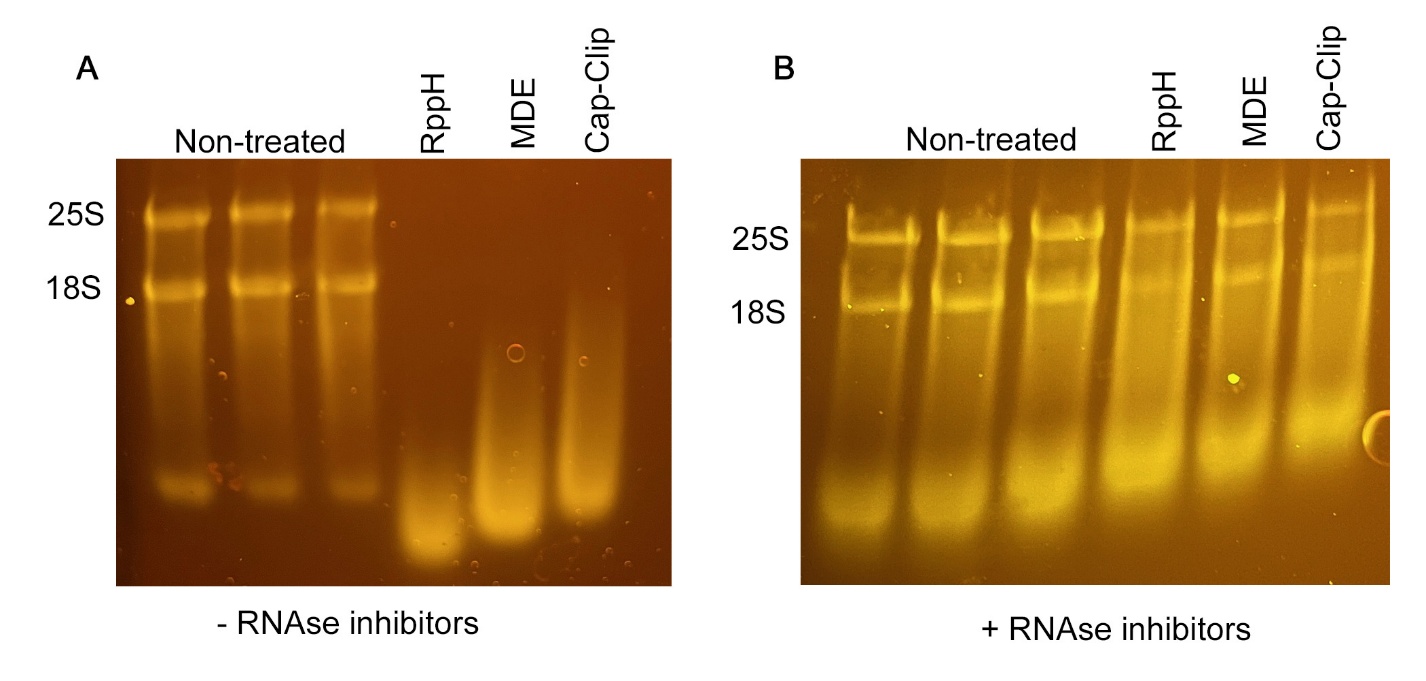


Supplementary Figure 2. RNA was treated with three different decapping enzymes: RppH, mRNA decapping enzyme (MDE) and Cap-Clip to check for endonuclease activity. First three lanes of the SYBR-gold-stained gels have non-treated RNA samples, while the next three lanes contain RNA samples that were digested by decapping enzymes. RNAse inhibitors were not used in (A), whereas in (B) RNA was incubated with RNAse nhibitors when the decapping enzymes were used.
